# Prevalence of Substance Use among Undergraduate Students in a Medical College of Nepal

**DOI:** 10.31729/jnma.4605

**Published:** 2019-10-31

**Authors:** Ramayan Prasad Kushwaha, Gajendra Prasad Rauniar, Bhawesh Koirala, Namita Kumari Mandal

**Affiliations:** 1 Department of Clinical Pharmacology and Therapeutics, BP Koirala Institute of Health Sciences, Dharan, Nepal

**Keywords:** *alcohol*, *diazepam*, *energy drinks*, *marijuana*, *tobacco*

## Abstract

**Introduction:**

The consumption of substances is a common practice among the medical students and their use might reduce educational and clinical performances as well as judgment of the students. This study aims to find out the prevalence of substance use among medical students in BP Koirala Institute of Health Sciences.

**Methods:**

A descriptive cross-sectional study was carried out among medical students of BP Koirala Institute of Health Sciences from 15^th^ September 15^th^ to December 15^th^ 2018 after obtaining ethical approval from the Institutional Review Committee (Ref: 1394/017). The study was conducted among 326 medical and dental students from first to fifth year by using the stratified sampling method. A self-reported questionnaire was developed which included types, frequency, duration, age to first use, and motives for the use of different substances. Data was analyzed using Statistical Package for Social Sciences version 11.5.

**Results:**

The prevalence of substance use among medical students of BP Koirala Institute of Health Sciences was 196 (61.4%) at 95% Confidence Interval (56.05-66.75%). Among substance use, the use of alcohol 190 (59.6%), tobacco 90 (28.2%), and marijuana 38 (11.9%) was more prevalent. One hundred forty four (45.2%) male students used more substances as compared to 52 (16.3%) female students. Fun sake or partying 131 (68.9%) was the main motivation of the students to use substances.

**Conclusions:**

The overall substance use among medical students was high compared to other studies. Alcohol was the most common substance misused by the student followed by tobacco and marijuana. Proper counseling and awareness programs about the potential risk of substances are recommended for the betterment of the students.

## INTRODUCTION

A sound mind is a continuous requirement of medical students for better education and patient care. Medical school life is an important transition period of life where students may start to consume alcohol, tobacco, and other substances. The use of these substances may cause several physical, psychological, emotional, and social problems in medical students.^1^

The use of addictive substances might cause psychiatric morbidity, cognitive and functional impairments, and behavioral changes that ultimately affect student health, wealth, and education. Medical students have a constant burden of academic and clinical activities and have easy access to drugs that might influence the students to misuse the substances.^2^ The importance of studies like this one is more as we lack the cumulative evidence related to substance use.

The study aims to find out the prevalence of substance use among medical students in BP Koirala Institute of Health Sciences.

## METHODS

A descriptive cross-sectional study was conducted at BP Koirala Institute of Health Sciences (BPKIHS), from September 15^th^ to December 15^th^, 2018. Ethical approval was obtained from the Institutional Review Committee of BPKIHS (IRC/1394/017). Prior to the study, students were informed about the objectives of the study and written consent was obtained. Inclusion criteria constituted all the students from the first year to the final year of both medical and dental disciplines. Students who did not agree to participate in the study or failed to give informed consent were excluded from the study. Confidentiality of the participants was maintained.

The sample size was calculated by using the following formula,

n = Z^2^ × p × (1-p)/

   = (1.96)^2^ × 0.425 (1-0.425)/(0.05)^2^

   = 375.52

   ≈ 376

where,

n = sample size
Z = 1.96 at 95% Confidence Interval
p = prevalence of substance use in BPKIHS, 42.5%.^3^
e = margin of error, 5%
Using the formula for finite population correction, s= n/(1+n/N)
The corrected sample size of the source population of 853 students was 376/(1+376/853) = 260.97
        ≈ 261.

After adding 20% non-response rate, the final sample size was 314. It was 36.8% of the total population. By using a stratified sampling technique, an equal proportion of male and female (36.8% each) students were selected by using the lottery method from both medical and dental disciplines, separately. However, the study was done among 326 students.

A self-prepared semi-structured questionnaire was used to conduct this survey, which was finalized after pre-testing on 30 students from different disciplines. The questionnaire mainly focused on the types, frequency, and duration, age to the first start, and motives for the use of different substances. It also included whether they started before or after joining the medical school and their use was increased or decreased after joining the BPKIHS. Those students who have used substances within 30 days were categorized as current substance users, and included in the study.^4^ Data were analyzed using SPSS version 11.5. Descriptive parameters such as frequency, mean, standard deviation, and percent were calculated and presented in table and graph.

## RESULTS

The prevalence of substance use among medical students in BP Koirala Institute of Health Sciences was 196 (61.4%) at 95% C.I. (56.05-66.75%). Alcohol 190 (59.6%) was the most common substance used by the students followed by tobacco 90 (28.2%), marijuana 38 (11.9%) and diazepam 3 (0.9%). Among 196 students, 144 (76.6%) male students used various substances as compared to 52 (26.5%) female students. Hence, the use of alcohol, tobacco and marijuana was more prevalent among male students (Table 1).

**Table 1:** Sex-wise distribution of substance use (n= 196).

Consumption pattern	Male, n (%)	Female, n (%)	Total, n (%)
Alcohol based products	64 (44.4)	39(75)	103 (52.5)
Tobacco based products	3(2.1)	3(5.7)	6(3.1)
Alcohol and Tobacco	39 (27.1)	07(13.6)	46 (23.5)
Alcohol, Tobacco and Marijuana	35 (24.3)	3(5.7)	38(19.4)
Alcohol and Diazepam	03 (2.1)	0 (0.0)	03(1.5)
Total	144(100)	52(100)	196(100)

**Figure 1:**
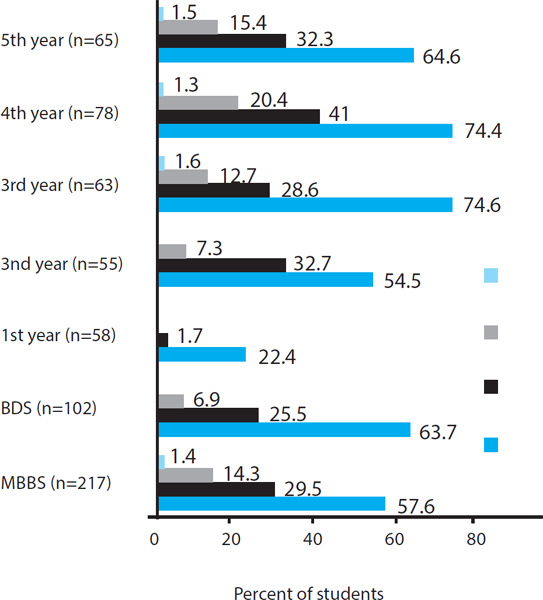
Study of year-wise distribution of substance use.

The majority of the students belonged to the medical background 217 (68%) while there were 102 (32%) students from dental background. The age of the participants ranged from 17-28 years (mean age of 21.76±1.81) among them majority have belonged to age group 20-25 years 281 (88.1%) (Figure 1).

**Figure:**
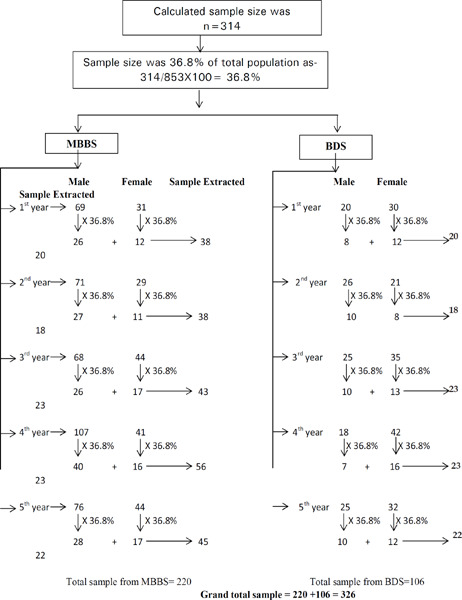


**Figure 2:**
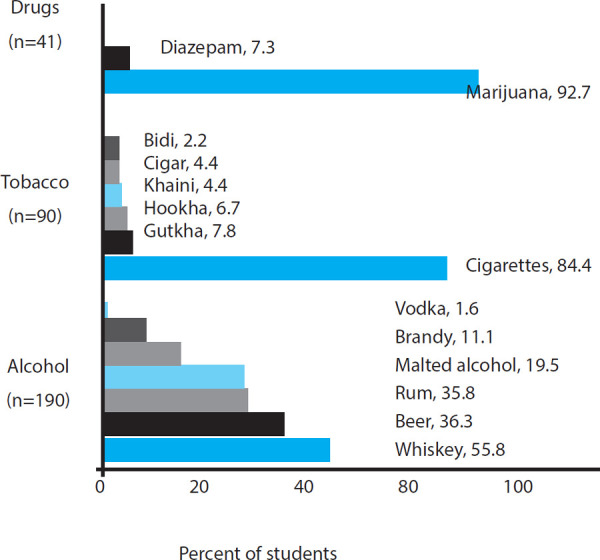
Types of substance used among the students.

Whisky 106 (55.8%), Wine 86 (55.3%), Beer 69 (36.3%), Cigarettes 76 (84.4%) and Marijuana 38 (92.7%) were the common substances used by the students (Figure 2).

## DISCUSSION

The overall prevalence of substance use among the medical and dental students of BPKIHS was 61.4%. A study conducted by Budhathoki, et al. on third-year medical students in Kathmandu valley found the overall prevalence of substance use to be 49.6%.^4^ Similarly, study by Khanal, et al. found 60.3% medical students had used various substances. Among them alcohol (57.6%), tobacco (27.6%), and cannabis (12.8%) were commonly used.^2^ These findings are well consistent with our survey in which the use of alcohol, tobacco, and marijuana was 59.6%, 28.2%, and 11.9% respectively. These patterns of the use of alcohol and tobacco might reflect the substance use pattern of Nepalese society and is recognized as a social sanction.^2^ However, the consumption of these substances is largely influenced by socio-demographic factors, education level, peer pressure, independent lifestyle, stressful activities, psychological factors and many more.^2-4^

Our study showed the use of alcohol (75%), tobacco (40.9%) and marijuana (18.6%) was more prevalent among male students as compared to females. Shyangwa et al. study in BPKIHS found 54.6% of male students drank alcohol.^3^ Khanal et al. study also depicted that the use of alcohol (70.6%), tobacco (39.2%), cannabis (20.8%) was more in males as compared to females.^2^ Similarly, Pitanupong, et al. study in Thailand showed 60% males and 48.1% females had drank alcohol.^5^ This male predominant use of substances was mainly due to our social structure that provides more freedom to male, and the male has a tendency to take more risk.

It is well reported that the progression of the educational ladder may influence the use of various substances. In our survey, the final year students used more substances as compared to first-year students. Medical students used more substances than dental students. Other studies also shown the students from different disciplines were used substances in various degrees. Panthee et al. found that the use of marijuana was common in pharmacy (13%) and public health students (10.6%) than nursing students (1.3%).^6^ Similarly, Shyangwa et al. found junior residents were the highest (72%) user of alcohol and lowest being undergraduate medical students (31.7%).^3^

A medical school is a vulnerable place where students may start to misuse various substances. Medical students have a burden full academic and clinical activity, easy access to substances that may catalyze the students to consume substances. In our study, 52.6% alcohol users, 56.6% tobacco users, and 82.9% marijuana users were stated to use these substances after joining the BPKIHS and their use of these substances was increased during the study period. Even, 61.1% of tobacco users consumed tobacco on a daily basis. One to four-time uses of alcohol and marijuana was 53.2% and 48.7% respectively. Khanal et al. also found 17.2% of medical students were stated to use substances after joining the medical school.^2^

Our survey showed that the main motives to use alcohol was fun sake or consumed during partying (68.9%), tobacco was to give pleasure and reduced stress or tiredness (64.4%) and the marijuana was friend encouragement to do it (65.9%). However, other studies showed the experimentation or curiosity followed by giving pleasure, stress relief, peer pressure were the main reasons for the use of substances.^2,4,7^

This study might help in education, prevention, screening, and policymaking related to the consequences of the substance use for the betterment of the students and the medical school environment. Although this study has several strengths, including the investigation of the motives and patterns of the use of substances, some limitations should be noted. The data of this study were based on the past 30-day’s memory, self-reported and drug-related, which may produce recall and response biases. The samples were medical and dental students of a medical college, so the finding of this study cannot be generalized to all Nepalese populations. A further study needs to be done to explore the amount and other factors that influence the use of substances.

## CONCLUSIONS

The overall substance use among undergraduate students was high. Alcohol was the most common substance misused by the student followed by tobacco and marijuana. Most of the students start their consumption of substances after joining medical school. Partying or fun sake, reduced stress or tiredness and friend encouragement were the motives for the use of the substances. Preventive measures such as proper counseling and awareness programs about the potential risk of substance use are further recommended for the betterment of the students.
